# Editorial: Neurocardiac Oscillation in Repolarization and Cardiac Arrhythmias

**DOI:** 10.3389/fphys.2020.604950

**Published:** 2020-10-27

**Authors:** Peter Taggart, George E. Billman

**Affiliations:** ^1^Department of Cardiovascular Sciences, University College London, London, United Kingdom; ^2^Department of Physiology and Cell Biology, The Ohio State University, Columbus, OH, United States

**Keywords:** autonomic nervous system, cardiac arrhythmias, ventricular repolarization, heart rate variability, action potential duration

## Introduction

Oscillations are a ubiquitous property throughout many biological systems. In the heart, beat to beat variability of heart rate (heart rate variability, HRV) and the ECG QT interval (QT variability, QTV) fluctuate over time at specific frequencies in particular, at a high frequency of about 0.25 Hz and at a lower frequency of about 0.1 Hz in humans (Task Force of the European Society of Cardiology North American Society of Pacing Electrophysiology, 1996; Billman, 2011; Baumert et al., 2016) These oscillations are related to the effect of the autonomic nervous system on the sinus node. High frequency (HF) oscillations of HRV occur at the respiratory frequency and are generally considered to reflect vagal activity and are widely used as a measure of parasympathetic activity. The physiological basis of low frequency oscillations (LF) of HRV is less clear cut but thought to reflect a combination of sympathetic and parasympathetic influence. The QT interval is a global representation of a combination of activation times and action potential duration (APD). As APD is strongly cycle length dependent, QTV is influenced by RR interval fluctuations as well as APD fluctuations particularly at faster heart rates when the APD falls on the steep part of its restitution curve. LF QTV has been shown to be increased by manoeuvers known to enhance sympathetic activity (Baumert et al., 2016) suggesting that at least a substantial part of the LF component of QTV may relate to sympathetic activity.

Both HRV and QTV have been shown to provide prognostic information in cardiac patients. Recently low frequency oscillations of ventricular repolarization measured from the ECG T wave vector, referred to as periodic repolarization dynamics, have been identified as one of the strongest predictors of sudden cardiac death (Rizas et al., 2014). These findings were confirmed and established for ventricular arrhythmia as well as sudden cardiac death in a large multicentre clinical trial (Bauer et al., 2019). These oscillations which are independent of respiration and enhanced during increased sympathetic activity have been proposed to relate to the effect of the intrinsic oscillation of sympathetic nerve activity on ventricular APD. In support of this contention, ventricular APD has recently been shown to exhibit oscillations at a low frequency in humans *in vivo* and these oscillations are enhanced by sympathetic provocation (Hanson et al., 2014; Porter et al., 2018). Potential cellular mechanisms underlying sympathetically mediated oscillations of ventricular APD have recently been identified (Pueyo et al., 2016).

Given the importance of oscillatory behavior in the clinical setting for both risk stratification and the identification of mechanisms for arrhythmogenesis, it is purpose of the present book to evaluate the physiology and electrophysiology of oscillatory behavior in the heart, particularly in the low frequency range. A particular focus has been placed on the oscillatory properties of ventricular repolarization and related aspects as briefly summarized as follows.

In chapter 1, Sprenkeler et al. using monophasic action potentials in a canine model showed that ventricular APD oscillated in both the HF and LF ranges. These oscillations were increased following remodeling induced by A-V block. LF power was greater in dogs in which Torsades de Pointes could be induced following dofetilide compared to non-inducible dogs. HF power was not related to inducibility (Sprenkeler et al.). In chapter 2, Palacios et al. describe novel methods to measure oscillations of ventricular repolarization from the ECG T-wave vector using continuous wavelet transform and phase rectified signal averaging. Microgravity was simulated by head down bed rest. Sympathetic stimulation using head up tilt increased oscillations before microgravity and more so following microgravity (Palacios et al.). In chapter 3, Van Duijvenboden et al. report the results of studies in patients showing that -adrenergic receptor blockade reduced LF APD oscillations and beat-to-beat APD variability. The two effects were correlated suggesting an interaction between the two. In chapter 4, computational modeling studies by Sampedro-Puente et al. revealed a time delay in the development of LF oscillations of APD following sympathetic stimulation. The mechanism was related to the slow phosphorylation kinetics of the slow component of the delayed rectifier current (Iks). In chapter 5, Orini et al. evaluated the effect of emotion (the response to pleasant or unpleasant music) on QT interval variability. These authors report that QT variability increased and was highly correlated with RR variability (Orini et al.). Since changes in RR interval elicit corresponding changes in QT interval, these results further confirm that QT variability should be measured during a constant RR interval (i.e., during atrial pacing, in order to obtain an accurate assessment of oscillations in ventricular repolarization independent of changes in RR interval). Chapter 6, reports the results of studies of HRV using a range of analytical methods showed a unique non-linear pattern in dogs compared to humans. These authors suggest that linearity was related to sympathetic dominance and non-linearity to parasympathetic dominance (Moïse et al.). In chapter 7, De Maria et al. addressed the much debated issue of the relative contributions of sympathetic and parasympathetic activity to the HF and LF components of HRV. They tested the combination of HF HRV in combination with LF QT variability as a measure of parasympathetic and sympathetic activity, respectively (De Maria et al.). They report that QT variability (SD) at rest identified the elderly patients with aortic stenosis who were at a greater risk of ventricular arrhythmia. In chapter 8, the effect of mental stress on the RR interval spectral power are discussed. Specifically these authors report that both LF and LF/HF spectral power increase in response to mental stress (Piccirillo et al.). In chapter 9, Ang and Marina evaluate the scientific evidence to identify neuronal networks responsible for generating LF rhythms along the neurocardiac axis. The functional significance of rhythmic sympathetic activity on neurotransmission efficiency and its role in the pathogenesis of repolarization instability is discussed. Finally in chapter 10, Schwartz and colleagues provide a personal overview of specific aspects of autonomic nervous system based on many years of their own pioneering research. These include the role of the baroreceptors, risk stratification, interventions to reduce sympathetic or enhance vagal nerve activity, RR and QT intervals (La Rovere et al.).

## Author Contributions

PT and GB jointly wrote the article, and proofread and approved submission of the article. All authors contributed to the article and approved the submitted version.

## Conflict of Interest

The authors declare that the research was conducted in the absence of any commercial or financial relationships that could be construed as a potential conflict of interest.
